# Improvement in Tongue Pressure Precedes Improvement in Dysphagia in Dermatomyositis

**DOI:** 10.3390/clinpract12050083

**Published:** 2022-09-29

**Authors:** Tomoo Mano, Shigeto Soyama, Kazuma Sugie

**Affiliations:** 1 Department of Rehabilitation Medicine, Nara Medical University, 840 Shijo-Cho, Kashihara 634-8521, Nara, Japan; 2 Department of Neurology, Nara Medical University, 840 Shijo-Cho, Kashihara 634-8521, Nara, Japan

**Keywords:** dermatomyositis, dysphagia, subjective evaluation, tongue pressure, videofluoroscopy

## Abstract

Dysphagia is known to occur in patients with dermatomyositis. However, the sudden-onset dysphagia without other symptoms can make diagnosis and treatment challenging. Two patients who did not have a severe muscle weakness complained of the sudden inability to swallow solids and liquids. The muscle biopsy results showed the perifascicular atrophy, and the patients were diagnosed with dermatomyositis. Videofluoroscopy revealed an inadequate pharyngeal contraction and a decreased upper esophageal sphincter opening with silent aspiration. Both patients showed low tongue pressures. Patient 1 received intravenous and oral methylprednisolone, and patient 2 received intravenous immunoglobulin in addition to intravenous and oral methylprednisolone. Several months after the onset of the dysphagia, the swallowing function of both patients improved. The improvement in tongue pressure preceded an improvement in the subjective and objective measurements of dysphagia. In conclusion, tongue pressure may be useful for predicting early improvement in swallowing function.

## 1. Introduction

Dermatomyositis, a systemic inflammatory disorder, affects various organs, including the skeletal muscles [1]. Dysphagia occurs in 18–20% of patients with dermatomyositis [2,3]. Patients with dermatomyositis should undergo a dysphagia evaluation irrespective of the limb involvement or dysphagia severity because the involvement of the muscles that are responsible for swallowing is unrelated to these factors [4]. The severe dysphagia of liquids, solids, and even saliva without the occurrence of a severe limb weakness is uncommon in dermatomyositis [5]. In these cases, no indicators of the effectiveness of treatment have been established. Surrogate markers that can be easily evaluated longitudinally at the bedside are necessary to search for effective treatment for each patient.

Patients with dermatomyositis with dysphagia experience tongue weakness, poor palatal motion, and the pooling of secretions in the distended hypopharynx. The swallowing dysfunction in dermatomyositis causes diffuse swallowing dysfunction, such as tongue weakness, flaccid vocal cords, and poor palatal motion. The tongue plays an important role in the oral stage of swallowing, which includes bolus formation and transportation [6].

We longitudinally evaluated the clinical course of two patients who presented with erythroderma and subsequently developed progressive dysphagia. To examine the responsiveness of them to treatment, we evaluated their swallowing function, using measures of tongue pressure, videofluoroscopy (VF), and a questionnaire. VF with a modified Logemann protocol was recorded the after participants were instructed to swallow 3 mL of 40% *w*/*v* barium sulfate twice in a standing position, which was viewed in the lateral plane [7]. Each swallowing activity was recorded, and the recording included at least 20 s of the period after the initial swallow, at a speed of 30 frames per second, using a digital capture card. The recorded images were analyzed frame-by-frame and scored based on the penetration-aspiration scale (PAS) and the pharyngeal residues were measured by using semiquantitative scales (percentage of residual volume in 3 mL): 0, 2, 5, 10, 20, 30, 40, 50, 60, 70, 80, 90, and 100%. The VF data were recorded using a Mini DV videotape (Sony, Tokyo, Japan) at 30 frames/s, and then two experienced speech-language pathologists verified these scales. The evaluation of the tongue function was often quite subjective, though the range of motion, strength, and coordination were basically used for the clinical evaluation of the swallowing function. However, it become clearer for the dysphagia patients to be able to understand the objective assessments and treatment goals including changing their tongue performance during swallowing and speech after the development of the tongue pressure measurement devices. We used a digital tongue pressure measurement device (TPM-01) (JMS Co., Ltd., Hiroshima, Japan), which has been approved for its use in Japan. The previous study reported that the maximum tongue pressure that was measured by the internationally used tongue pressure measurement, such as the Iowa Oral Performance Instrument (IOPI), was slightly higher than that which was measured by the TPM-01 [8,9].

The two patients compressed the balloon of a disposable intraoral pressure probe upward onto their palates for 7 s using the maximum voluntary effort of the tongue. The tongue pressures were recorded three times at 1-min intervals and we adopted the maximum tongue pressure (kPa). We used the average of the maximal tongue pressures for our evaluation [10].

## 2. Case Presentations

### 2.1. Case 1

**Figure 1:**
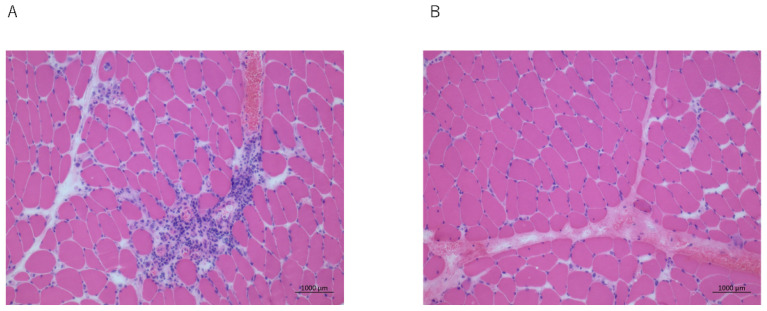
Pathological findings. Photomicrographs of the muscle biopsy specimens. (**A**) Features of inflammatory myopathy, such as focal perifascicular atrophy and perivascular lymphocytic infiltration of the perimysium and endomysium, are seen (patient 1); (**B**) Features of inflammatory myopathy are seen (patient 2). Scale bar = 150 μm (**A**,**B**).

A 28-year-old Asian man presented with a 7-month history of progressive swallowing difficulty and erythroderma. His proximal and distal muscle strengths were of grade 4 and grade 5 (Medical Research Council scale), respectively. The respiratory, cardiovascular, and abdominal examinations revealed that there were no abnormalities. A poor laryngeal elevation on palpation and a bilaterally decreased gag reflex were noted. The inflammatory marker levels were elevated: the erythrocyte sedimentation rate was 110 at 1 h after presentation, and the C-reactive protein (CRP) level was 45 mg/L. The creatine phosphokinase (630 U/L, normal: 26–140 U/L) and lactate dehydrogenase (712 U/L, normal: 225–450 U/L) levels were elevated. The antinuclear antibody positivity (1:400, speckled staining pattern) and anti-Jo antibody negativity levels were noted on the immunohistochemical examination of the muscle biopsy specimens. Electromyography revealed short-amplitude, polyphasic, small motor unit potentials with early recruitment. The features of inflammatory myopathy, such as a focal perifascicular atrophy and the perivascular lymphocytic infiltration of the perimysium and endomysium, and some degenerating and necrotic muscle fibers with mild chronic inflammatory cell infiltrates, were noted on the quadriceps muscle biopsy (Figure 1A). The VF revealed that there was residue in the valleculae and pyriform sinuses, the PAS was 3, and the percentage of the pharyngeal residues was 10%. The patient’s tongue pressure was 15.7 kPa. The Eating Assessment Tool-10 score was 9/40. Magnetic resonance imaging revealed that there were no swallowing muscle abnormalities.

**Figure 2:**
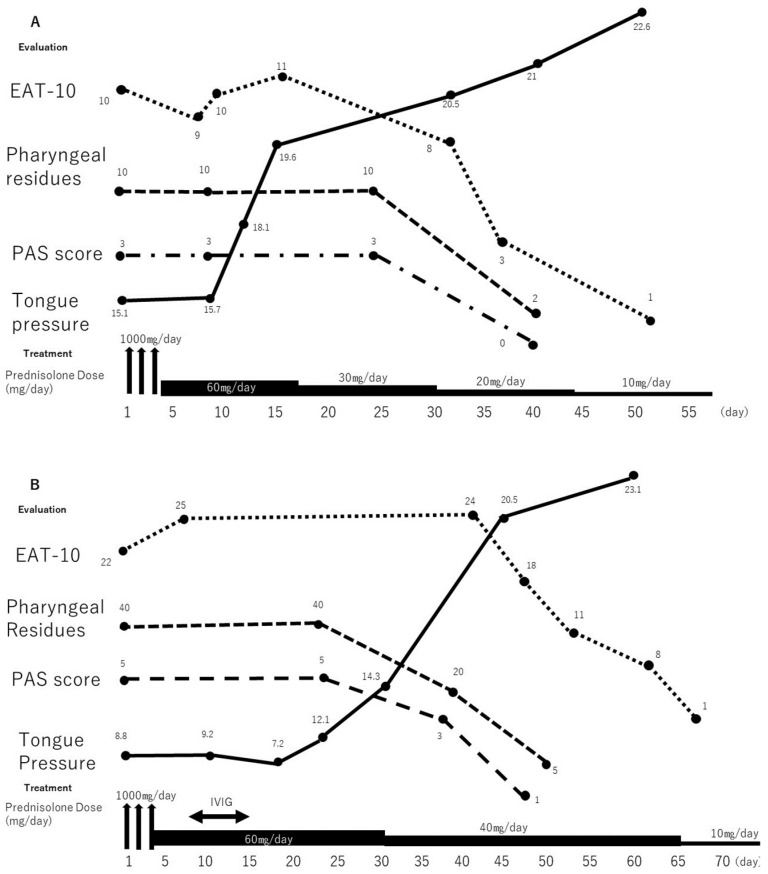
Timeline of the clinical course of the patients. (**A**) Changes in the results of swallowing evaluations and treatment provided in case 1 are shown. Tongue pressure improved slightly with prednisolone therapy. Subsequently, improvements were noted on VF and subjective evaluation. (**B**) Changes in the results of swallowing evaluations and treatment provided in case 2 are shown. Swallowing function and tongue pressure showed no improvement despite pulse treatment with 1000 mg of methylprednisolone. Therefore, additional treatment with intravenous immunoglobulin was administered. One week later, the tongue pressure improved slightly, followed by improvement on VF and subjective evaluation. EAT-10: Eating Assessment Tool-10, IVIG: intravenous immunoglobulin, PAS: Penetration-Aspiration Scale, VF: videofluoroscopy.

After a pathological diagnosis, a methylprednisolone pulse treatment (1000 mg) was initiated, and it was tapered to 40 mg/day of prednisolone. The patient underwent a subjective dysphagia assessment and a tongue pressure measurement weekly and a VF every 2 weeks. After the methylprednisolone pulse therapy was initiated, the serum creatine kinase level was 574 U/L, and muscle strength improved to grade 5. Tongue pressure improved after 1 week, but the subjective dysphagia did not. After 3 weeks, the VF findings and the subjective dysphagia improved. The clinical course is shown in Figure 2A. 

### 2.2. Case 2

A 56-year-old woman presented with a 3-month history of progressive swallowing disturbances. Two weeks later, her dysphagia worsened, and she experienced hypernasal speech. Her limb and neck flexor muscle strengths were of grade 5 and grade 4, respectively. She could not swallow liquids, solids, or even saliva. A poor laryngeal elevation was noted on the palpation, and she was initiated on tube feeding after her admission. She had a CRP level of 5.0 mg/L, a creatine phosphokinase level of 1126 U/L (elevated), and anti-Jo antibody negativity. The features of the inflammatory myopathy, such as a focal perifascicular atrophy, were observed on the biceps brachii muscle biopsy (Figure 1B). She was diagnosed with dermatomyositis. The VF revealed that the PAS was 6, and the percentage of the pharyngeal residues was 40%. The methylprednisolone pulse treatment (1000 mg) was initiated, and it was tapered to 40 mg/day of prednisolone; however, the swallowing function and tongue pressure showed no improvement. Therefore, the patient received intravenous immunoglobulin (IVIG, 400 mg/kg/day) for five consecutive days. No rapid improvement in the dysphagia occurred, and she was administered intravenous methylprednisolone (1 mg/kg/day) and received swallowing rehabilitation. One week later, her tongue pressure improved slightly, although the subjective dysphagia assessment findings did not change. Two months after the dysphagia onset, she was able to swallow saliva. She was treated with oral prednisolone (40 mg/day for 2 weeks). The follow-up VF demonstrated that there was an improved pharyngeal contraction and decreased pharyngeal residues (Figure 2B). There was no aspiration when she was swallowing a puree or silent aspiration when she was swallowing a liquid. Therefore, oral feeding with a level 2 dysphagia diet (pureed foods with honey-like fluids) was started [8]. She was discharged after 2 months of hospitalization; at that time, she was receiving oral prednisolone (20 mg/day). Throughout the treatment period, she underwent swallowing rehabilitation, which included compensatory maneuver training and oropharyngeal exercises. In the VF examination that was 3 months after dysphagia onset, the pharyngeal residues decreased, and she started receiving a regular diet without difficulty. The clinical course is shown in Figure 2B. 

## 3. Discussion

We reported two cases of patients who experienced a sudden-onset aphagia without having severe limb weaknesses, and were monitored for their treatment responsiveness and pathological control [11]. Dermatomyositis is primarily treated with corticosteroids. Our patients received multiple treatments owing to their life-threatening conditions. Dysphagia, which is a risk-factor of malignancy comorbidities, is much more common in patients with steroid-resistant dermatomyositis than in those with steroid-responsive dermatomyositis. Steroid-resistant dysphagia may respond to high-dose IVIG [12]. IVIG has been used in patients with dermatomyositis who are refractory to steroid-sparing treatments. IVIG blocks the Fc receptors on the vascular wall, thereby preventing membrane attack complex deposits from entering the endomysial capillaries. Positive effects on myopathy and cutaneous ulceration have also been reported [13]. 

Neither of our patients had malignancy comorbidities, but they showed immediate improvement in their dysphagia. Moreover, patient 2 demonstrated a complete improvement of life-threatening steroid-resistant dysphagia. In general, the treatment response with muscle weakness was evaluated using the following parameters: serum creatine kinase level and muscle strength [12]. However, the patients with only the dysphagia could not be monitored, and therefore, it is difficult to determine whether the treatment that was provided was effective. However, a subjective evaluation may not reliably indicate the swallowing status in patients with myopathy or adequately detect any early changes. We need the swallowing evaluation marker for the efficacy of the treatment at the bedside. 

The exact pathophysiology underlying myogenic dysphagia is not fully understood, and it is subject of controversial discussions. The intrinsic muscles of the larynx are phenotypically similar to the limb skeletal muscles, expressing type I (slow), and IIa and IIx (fast) fibers, with additional specialized isotypes such as IIL (superfast) and slow tonic fibers [11,14]. Thus, it is plausible, if not likely, that the autoantigen(s) that is/are responsible for the immune attack on the limb skeletal muscle are also present on the myocytes or endomysial capillaries of the laryngeal musculature. A recent study suggested that dysphagia in patients with inflammatory myopathy appears to mostly be due to impaired pharyngeal muscle contraction stemming from suprahyoid muscle weakness rather than failed upper esophageal sphincter relaxation, which is a commonly implicated mechanism [3,15]. Taken together, the swallowing function evaluation of myositis requires not only the pharyneal and laryngeal, but also the oral cavity. 

Swallowing rehabilitation techniques are more applicable in patients with pharyngeal motility dysfunction than in those with esophageal motility dysfunction [7]. The VF can reveal the status of the swallowing and the presence and severity of dysphagia [16]. Two months after the dysphagia onset, patient 2 showed a complete clinical recovery, but the VF revealed that there was a silent aspiration with liquids. Therefore, the VF is considered to be a more sensitive test method than an assessment of the subjective symptoms is. Indeed, many studies have reported that routine videofluoroscopy followed the clinical course of swallowing. On the other hand, a VF should be performed despite the disadvantages of radiation exposure, allergic reaction, and the requirement of the patient to visit the radiography room [7]. Fiberoptic Endoscopic Evaluation of Swallowing (FEES) is another option for a semi-quantitative and precise swallowing evaluation, and it can be performed at the bedside. However, the FEES operator requires training, experience, and skill, and there is a possibility of bias occurring. On the other hand, tongue pressure also plays an important role in the swallowing function. Tongue pressure is measured using an intraoral pressure probe; this technique is non-invasive, simple, and does not require training, and it can be performed even at the bedside. It was previously reported that tongue pressure is strongly correlated with the scores of the questionnaires regarding swallowing, and it can reflect the dysphagia severity in patients with neuromuscular diseases. The VF provides much more valuable information than tongue pressure does. However, tongue pressure may be used as a screening test at the bedside since dysphagia can be life-threatening and long-lasting. The VF was used to identify the suitable biomarker for the progress of the improvement or deterioration in neuromuscular disease [16,17,18], and tongue pressure measurement might also be a useful method for monitoring the effect of treatments and swallowing rehabilitation [19]. 

## 4. Conclusions

We report two cases of a sudden-onset severe dysphagia due to the exacerbation of underlying dermatomyositis without the aggravation of other symptoms. A multidisciplinary approach is necessary to rule out the structural and neurological causes of acute dysphagia. A detailed dysphagia evaluation is important as it enables the appropriate treatment selection, and the appropriate medical and rehabilitative treatment should be provided because, in patients with dermatomyositis, their recovery may take months.

## Data Availability

The data presented in this study are available on request from the corresponding author.
